# Linking growth mindset to engagement via ideal L2 self and enjoyment in AI-mediated EFL writing: a mixed-methods study

**DOI:** 10.3389/fpsyg.2026.1836353

**Published:** 2026-06-16

**Authors:** Hongbin Li, Shengxuan Geng

**Affiliations:** School of Foreign Languages, Tianjin University, Tianjin, China

**Keywords:** AI-mediated EFL writing, engagement, enjoyment, growth mindset, ideal L2 self

## Abstract

This study investigates the psychological mechanisms underlying engagement in AI-mediated EFL writing from a social cognitive theory perspective. Specifically, it examines how growth mindset, ideal L2 self, and enjoyment relate to engagement. An explanatory sequential mixed-methods design was employed. In the quantitative phase, survey data were collected from 519 university EFL learners from ethnic minority regions in China and analyzed using structural equation modeling. In the qualitative phase, semi-structured interviews were conducted with 20 participants to elaborate on learners’ experiences and explain the quantitative results. The quantitative findings revealed significant positive associations among growth mindset, ideal L2 self, enjoyment, and engagement in AI-mediated EFL writing. Mediation analyses further indicated that growth mindset was indirectly related to engagement through ideal L2 self and enjoyment, including both single-mediator pathways and a chained pathway via ideal L2 self followed by enjoyment. The qualitative findings further showed that learners maintained engagement by reframing challenges and feedback as learning opportunities, connecting writing to future self-guides, and experiencing enjoyment during iterative revision. This study advances understanding of engagement in AI-mediated EFL writing by highlighting the interplay between personal beliefs, motivational self-guides, and affective experiences, and it offers practical implications for designing AI-supported writing instruction that fosters sustained learner involvement.

## 1 Introduction

The increasing integration of artificial intelligence (AI) into second language (L2) writing has fundamentally reshaped how learners, particularly those from ethnic minority regions in China, plan, draft, and revise their texts (Barrot, 2023; Gao and Wang, 2026; Wang and Wang, 2024). AI-mediated writing tools, such as automated feedback systems and large language models, provide learners from ethnic minority regions in China with immediate linguistic support and revision suggestions, creating new opportunities for writing development in EFL contexts, especially in areas where resources may be limited (Alghasab, 2025). A growing body of research has examined the effects of AI-mediated writing tools on learners’ writing performance, revision quality, and attitudes toward technology use (Bekdaş, 2026; Hartshorn and Pack, 2026).

However, much of this research has focused primarily on mainstream EFL contexts (Duong and Chen, 2025; Shi et al., 2025; Wang X. et al., 2024), offering limited insight into learners’ psychological experiences during AI-mediated EFL writing. This limitation is particularly notable for ethnic minority EFL learners in China, whose English writing experiences may be shaped by distinctive educational, linguistic, sociocultural, and technological conditions (Jiang et al., 2020). Educationally, some ethnic minority learners may have relatively uneven access to high-quality English writing instruction, individualized feedback, and academic language support, especially in resource-limited regions (Zhang et al., 2025). Linguistically, many of them negotiate multilingual learning experiences involving their home languages, Mandarin Chinese, and English, which may influence how they understand writing tasks, process AI-generated feedback, and develop confidence in English expression (Yang and Liang, 2025). Socioculturally, English writing may be connected with identity negotiation, academic mobility, and future participation in wider educational or professional communities (Wang and Guo, 2026). Technologically, AI-mediated writing tools may provide immediate feedback and additional support, but learners’ ability to benefit from these tools may depend on digital access, AI literacy, prior writing experience, and teacher guidance. Therefore, examining engagement in AI-mediated EFL writing among ethnic minority learners can provide a more context-sensitive understanding of how learners respond to AI-supported writing environments.

To address this gap, the present study examines the psychological processes underlying engagement in AI-mediated EFL writing among ethnic minority EFL learners in China. Rather than claiming novelty in the constructs themselves, this study contributes by examining how growth mindset, ideal L2 self, and enjoyment are sequentially associated with engagement in this specific context. Previous studies have examined related motivational and emotional pathways in broader L2 learning contexts, but less is known about how these psychological factors operate together when learners engage in AI-supported writing and iterative revision. In this study, growth mindset is positioned as a belief-related factor, ideal L2 self as a future-oriented self-guide, and enjoyment as an affective experience, with engagement as the focal learning outcome. By situating this chained mediation model in AI-mediated EFL writing and grounding it in the experiences of ethnic minority learners, the study aims to clarify how personal beliefs, future self-guides, and positive emotions jointly support sustained writing engagement in a technology-rich learning environment.

## 2 Literature review

### 2.1 AI-mediated EFL writing

With the growing integration of AI in language education, EFL writing is increasingly situated in AI-mediated environments where learners’ composing is shaped by AI-generated feedback and revision suggestions (Wang et al., 2026; Wang et al., 2025). Unlike traditional writing, AI-mediated EFL writing features iterative drafting, immediate feedback, and sustained learner–technology interaction, reshaping how writing is learned and practiced (Chen and Gong, 2025; Gao and Wang, 2022; Giglio and Costa, 2023). In these settings, writing becomes an ongoing, AI-mediated meaning-making process across multiple stages of composition rather than a one-off, product-oriented task (F. Sun et al., 2025; Wang and Wang, 2025). Learners must not only respond to AI feedback but also evaluate, select, and integrate suggestions into their texts, increasing the demands for active involvement (Dergaa et al., 2023; Fang et al., 2023). Accordingly, engagement has become a central concern in AI-mediated EFL writing, as meaningful learning depends on sustained behavioral, cognitive, and emotional participation across repeated drafting and revision cycles (Bekdaş, 2026; Hartshorn and Pack, 2026; Tang et al., 2026). Identifying factors that facilitate or hinder engagement is therefore essential for explaining how AI technologies contribute to EFL writing (Gao et al., 2025a; Wang and Reynolds, 2024).

Previous research on AI-mediated EFL writing has approached this emerging learning context from multiple perspectives. First, a substantial body of studies has examined the effects of AI-mediated writing tools on learners’ writing performance, focusing on text quality, linguistic accuracy, and revision outcomes, with findings generally suggesting that AI-generated feedback can support drafting and revising processes, particularly at the surface level (Abdi Tabari et al., 2025; Luo and Yusuf, 2026). Second, another line of research has explored learners’ perceptions and attitudes toward AI-mediated EFL writing, addressing issues such as perceived usefulness, trust in AI feedback, and concerns about overreliance or academic integrity, and indicating that learners’ subjective evaluations of AI shape how actively they engage with writing tasks (Hartshorn and Pack, 2026; Sun et al., 2026b). Third, an increasing number of studies have situated AI-mediated EFL writing within broader pedagogical and instructional frameworks, highlighting the roles of task design, instructional scaffolding, and teacher mediation in shaping learners’ use of AI tools (Fathi and Rahimi, 2026). However, despite these advances, relatively little is known about the psychological mechanisms that sustain learners’ engagement in AI-mediated EFL writing over time. To address this gap, the present study adopts a psychological perspective to examine how learners’ growth mindset, ideal L2 self, enjoyment jointly shape engagement in AI-mediated EFL writing contexts.

### 2.2 Empirical studies on psychological factors and engagement in AI-mediated EFL writing

Previous studies have examined a range of factors associated with engagement, including learner beliefs, motivational variables, emotional experiences, and contextual support (e.g., Gao et al., 2025b; Shen et al., 2024; Sun et al., 2026a; Tang et al., 2026; Zhang et al., 2024). Among these factors, growth mindset has received increasing scholarly attention. Growth mindset refers to learners’ beliefs that their abilities are malleable and can be developed through sustained effort, strategic practice, and appropriate support (Lou and Noels, 2019). Accumulating evidence suggests that learners who hold a stronger growth mindset are more likely to form a clearer and more attainable ideal L2 self, as they tend to view improvement as feasible through continued effort and deliberate strategy use (Fathi et al., 2024; Yao et al., 2024). Relatedly, learners with a stronger growth mindset also tend to report higher enjoyment because they are more inclined to appraise challenges as manageable and to interpret feedback as supportive rather than threatening (Xu, 2025; Yang and Wu, 2025). Growth mindset has likewise been linked to engagement in foreign language learning contexts (Bai and Wang, 2023). Specifically, learners endorsing a stronger growth mindset are more likely to persist, invest greater effort, and adopt more adaptive learning strategies, which constitute key behavioral manifestations of engagement (Jiang et al., 2024; Vestad and Bru, 2024).

Although these associations are well documented in general L2 learning, evidence remains limited in skill-specific domains such as L2 writing, particularly in AI-mediated contexts. This gap is notable because AI-mediated EFL writing typically involves frequent automated feedback and iterative cycles of drafting and revision (Sun et al., 2026b), which place sustained demands on learners’ motivation, affect, and self-regulation. In this setting, a growth mindset may be especially relevant for sustaining engagement by normalizing iterative improvement and supporting continued strategic effort across revisions. Accordingly, the following hypotheses were proposed:

*H1*. Growth mindset is positively associated with ideal L2 self in AI-mediated EFL writing.

*H2*. Growth mindset is positively associated with enjoyment in AI-mediated EFL writing.

*H3*. Growth mindset is positively associated with engagement in AI-mediated EFL writing.

Ideal L2 self refers to learners’ envisioned future identity as competent and successful users of the second language (Ueki and Takeuchi, 2013). Within the L2 motivational self system, a stronger and more vivid ideal L2 self is typically associated with more positive emotional experiences and stronger motivated behavior (Wang et al., 2022). When learners perceive current learning activities as instrumental for approaching valued future goals, they tend to experience greater satisfaction and enjoyment (Fathi and Hejazi, 2024; Zhao et al., 2023). Likewise, a more vivid ideal L2 self has been linked to greater intended effort, persistence, and active participation, which are central indicators of engagement (W. Sun et al., 2024; Zhu et al., 2023). Extending these insights to AI-mediated EFL writing, an ideal L2 self may function as a salient self-guide that supports enjoyment and sustained engagement during feedback-driven revision. Accordingly, the following hypotheses were proposed:

*H4*. Ideal L2 self is positively associated with enjoyment in AI-mediated EFL writing.

*H5*. Ideal L2 self is positively associated with engagement in AI-mediated EFL writing.

Enjoyment is defined as a positive emotional experience characterized by feelings of pleasure, interest, and satisfaction during the learning process (Dewaele et al., 2018; Wang Z. et al., 2024). A substantial body of research in foreign language learning suggests that enjoyment is positively associated with engagement, reflected in greater behavioral participation, deeper cognitive investment, and stronger persistence in demanding language tasks (Liu, 2022; Ünsal-Görkemoğlu and Karacan, 2024). In AI-mediated EFL writing, enjoyment may be particularly important because it can energize learners’ willingness to re-engage with feedback and continue refining their texts across multiple revision rounds (Huang and Zhang, 2025; Jiang and Chen, 2026). Therefore, the following hypothesis was proposed:

*H6*. Enjoyment is positively associated with engagement in AI-mediated EFL writing.

### 2.3 Theoretical framework: social cognitive theory

Social cognitive theory (SCT) conceptualizes learning as a dynamic process shaped by reciprocal interactions among personal factors, behavioral processes, and environmental conditions (Bandura, 1986, 1997). This framework provides a useful lens for understanding AI-mediated EFL writing, where generative AI tools offer immediate feedback, suggestions, and opportunities for iterative revision. However, the present study does not aim to empirically test the full triadic reciprocity proposed by SCT. Rather, SCT is used as a guiding framework to explain how selected personal factors are associated with learners’ engagement within an AI-mediated writing environment. In this context, the availability of AI support alone may not ensure sustained engagement. Learners’ engagement may also depend on how they interpret AI-generated feedback, how they regulate effort across revision cycles, and how their beliefs, future self-guides, and emotions develop during AI-supported writing.

**Figure 1:**
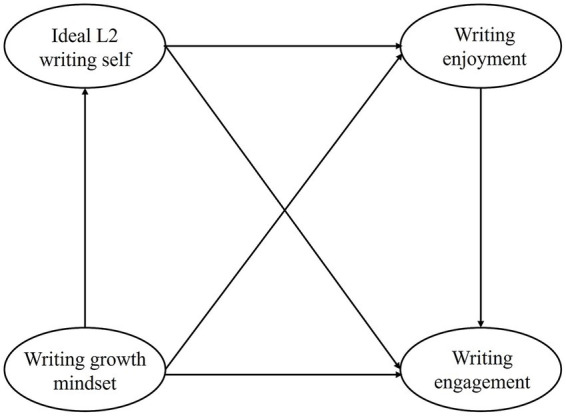
The proposed model.

In the present study, growth mindset, ideal L2 self, and enjoyment are conceptualized as key personal factors, while engagement is treated as the behavioral process and focal outcome. The AI-mediated writing context is understood as the broader learning environment in which these personal and behavioral processes occur, rather than as a directly measured variable in the model. Consistent with SCT (Bandura, 1986), the model assumes that learners’ personal factors may shape their engagement in AI-supported writing activities. Specifically, growth mindset is expected to be positively associated with engagement, both directly and indirectly through ideal L2 self and enjoyment. Therefore, this study investigates the interplay between personal and behavioral variables within an AI-mediated writing setting, rather than claiming to capture the complete SCT model. The hypothesized model is presented in Figure 1.

## 3 Methodology

### 3.1 Research design

This study employed an explanatory sequential mixed-methods design (Creswell and Clark, 2011). In the quantitative phase, a questionnaire survey was conducted to examine the associations among growth mindset, ideal L2 self, enjoyment, and engagement in AI-mediated EFL writing. Building on the quantitative results, a qualitative phase was subsequently implemented. Semi-structured interviews were used to explore learners’ experiences and perspectives and to provide deeper insight into how and why these associations occurred in AI-mediated EFL writing contexts. The integration of the two strands occurred mainly at the interpretation stage. Specifically, the qualitative findings were used to explain and elaborate on the statistically significant pathways identified in the SEM results, including the direct and indirect associations among the four focal variables. This strategy enabled the study to move beyond identifying statistical relationships and to interpret how learners experienced these psychological processes during AI-supported writing and revision. At the quantitative and qualitative phases, the study was guided by the following research questions:

*RQ1*: What are the associations among growth mindset, ideal L2 self, enjoyment, and engagement in AI-mediated EFL writing?

*RQ2*: How do learners explain their engagement in AI-mediated EFL writing?

### 3.2 Participants

A total of 519 ethnic minority students completed the online survey. Eligible respondents were university students from ethnic minority backgrounds who had prior experience using AI tools for English writing and participated voluntarily. In this study, AI-mediated EFL writing refers to students’ use of AI tools to assist English writing activities across formal and informal learning contexts, including classroom-based tasks and self-directed writing practices. The study focused on students’ general AI-supported English writing experiences rather than the effects of specific AI tools or instructional settings. Of the total sample, 151 (29.09%) identified as Zhuang, 120 (23.12%) as Uyghur, 155 (29.87%) as Hui, and 69 (13.29%) as Mongolian. The remaining 24 participants (4.62%) reported other ethnic backgrounds, including Manchu (n = 14, 2.70%) and Miao (n = 10, 1.93%). In terms of gender, 275 (52.99%) participants were male and 244 (47.01%) were female. Regarding age, 460 (88.63%) were between 18 and 21 years old, while 59 (11.37%) were 22 years old and above. In terms of academic discipline, 232 (44.70%) students were from the humanities and social sciences, 259 (49.91%) were from the natural sciences, and 28 (5.39%) reported other fields.

Additionally, 20 participants took part in follow-up semi-structured interviews. Interviewees were selected to reflect variation in ethnicity, gender, age, and disciplinary background. They included students from multiple ethnic minority groups and academic fields, both male and female students, and participants from the age groups represented in the survey. All had prior experience using AI tools for English writing.

### 3.3 Instruments

Data were collected using two instruments administered in Chinese: a structured questionnaire and follow-up semi-structured interviews. For the questionnaire, the original English scales were translated into Chinese through a translation and back-translation procedure. Two bilingual researchers independently translated the original English items into Chinese and then independently back-translated the Chinese version into English. The original, translated, and back-translated versions were compared, and any discrepancies were discussed and resolved by the research team. The final Chinese version was further reviewed by two experts in applied linguistics and EFL teaching to ensure semantic equivalence, clarity, and contextual appropriateness for ethnic minority EFL learners. The questionnaire contained a demographics section (e.g., age, gender, and ethnicity) and a measures section targeting four focal variables: growth mindset, ideal L2 self, enjoyment, and engagement. Responses were recorded on a 5-point Likert scale ranging from 1 (strongly disagree) to 5 (strongly agree). In addition, a semi-structured interview guide was designed to elicit participants’ experiences and perceptions of AI-supported EFL writing.

#### 3.3.1 Growth mindset

Growth mindset was measured using the three-item Growth Mindset Scale (Yao et al., 2021). All items were adapted to the context of AI-supported EFL writing. A sample item was “No matter who I am, I can significantly improve my English writing skills with the support of AI tools.” The measure showed good internal consistency in this study (Cronbach’s *α* = 0.872).

#### 3.3.2 Ideal L2 self

Ideal L2 self was assessed using the six-item Ideal L2 Writing Self Scale (Tahmouresi and Papi, 2021). All items were adapted to the context of AI-supported EFL writing. A sample item was “I can see a day when I can write in English fluently and accurately with the help of AI.” In this study, the measure showed good reliability (Cronbach’s α = 0.883) and model fit (*χ^2^/df* = 2.194, RMSEA = 0.048, NFI = 0.986, RFI = 0.977, IFI = 0.993, TLI = 0.987, CFI = 0.992).

#### 3.3.3 Enjoyment

Enjoyment was measured with four items from the enjoyment subscale of the Achievement Emotions Questionnaire (Bieleke et al., 2021). All items were contextualized for AI-supported EFL writing. A sample item was “When my English writing is going well with the support of AI tools, it gives me a rush.” The measure showed excellent internal consistency (Cronbach’s α = 0.865) and acceptable model fit (*χ^2^/df* = 4.162, RMSEA = 0.078, NFI = 0.991, RFI = 0.974, IFI = 0.993, TLI = 0.980, CFI = 0.993) in this study.

#### 3.3.4 Engagement

Engagement was assessed using the nine-item Language Classroom Engagement Scale (Eerdemutu et al., 2024), with item wording adapted to reflect AI-mediated EFL writing. The measure captures three facets of engagement, behavioral, emotional, and cognitive, each represented by three items. A sample item was “I enjoy learning new things about writing in AI-empowered English writing.” In this study, the scale showed good reliability, with Cronbach’s α = 0.850 for behavioral engagement, 0.867 for emotional engagement, and 0.874 for cognitive engagement, and 0.884 for the total scale. The scale also showed excellent model fit (*χ^2^/df* = 1.225, RMSEA = 0.021, NFI = 0.989, RFI = 0.983, IFI = 0.998, TLI = 0.997, CFI = 0.998).

#### 3.3.5 Semi-structural protocol

The semi-structured interview protocol was developed to explore how learners explain their engagement in AI-mediated EFL writing. Drawing on the quantitative model and the key constructs examined in this study, the interview questions were designed to elicit learners’ experiences with challenges and feedback, their perceptions of themselves as current and future English users, and their emotional experiences during AI-supported EFL writing and revision. Rather than directly asking participants about abstract constructs, the questions encouraged learners to describe concrete writing experiences, typical reactions, and personal reflections. This approach allowed participants to articulate how they interpret feedback, how they connect writing activities to their future goals, and how they experience enjoyment or frustration in AI-mediated writing.

### 3.4 Data collection

Quantitative data were collected in January 2026 through an online survey using convenience sampling. The survey link (Wenjuanxing) was distributed by university instructors and circulated on social media platforms, including Xiaohongshu and QQ. Although recruitment emphasized participation from ethnic minority students, the open online distribution meant that both Han and ethnic minority students could access and complete the questionnaire at the initial stage. In line with the focus of the present study, respondents who identified as Han were excluded during data screening. A total of 867 responses were received. After excluding 308 responses from Han students (35.52%) and 40 additional invalid cases (4.61%), such as responses with very short completion times or invariant response patterns, the final analytic sample consisted of 519 valid questionnaires (59.86%).

For the qualitative phase, one-on-one semi-structured interviews were conducted to further explore the survey results. Respondents had the option to provide contact details at the end of the questionnaire. From this pool, 30 individuals were purposively selected based on their responses, and 20 participated after considering non-responses and withdrawals. The interviews, conducted in Chinese via Tencent Meeting, lasted approximately 30–50 min each and were audio-recorded for verbatim transcription and subsequent thematic analysis.

### 3.5 Data analysis

Quantitative analyses were carried out in SPSS 27.0 and AMOS 26.0. First, the measurement model was evaluated through confirmatory factor analyses (CFA). Second, common method bias was assessed using Harman’s single-factor test, and multicollinearity was examined using variance inflation factor (VIF) values. Distributional assumptions were then assessed using skewness and kurtosis, followed by descriptive statistics and Pearson correlation analyses. Finally, the hypothesized structural relations were tested using SEM, and indirect effects were estimated via bootstrap resampling.

For the qualitative strand, interview transcripts were managed and coded in MAXQDA 24 using reflexive thematic analysis (Braun and Clarke, 2006). Analysis proceeded through iterative cycles of transcript familiarization, coding, and theme development. The resulting themes were integrated with the quantitative results to explain and elaborate on the observed statistical patterns, highlighting contextual and individual factors that may help account for the associations identified in the survey.

## 4 Results

### 4.1 Quantitative results

#### 4.1.1 Results of CFA

As reported in Table 1, the four constructs demonstrated satisfactory psychometric properties. Composite reliability (CR) values exceeded 0.70 and average variance extracted (AVE) values were above 0.50, providing evidence of internal consistency and convergent validity (Hair et al., 2021). Discriminant validity was supported because the square root of AVE for each construct was greater than its correlations with the other constructs (Fornell and Larcker, 1981).

**Table 1:** Results of reliability and validity checks.

Variable	CR	AVE	The square root of AVE and correlation coefficient matrix			
			1	2	3	4
1 Growth mindset	0.872	0.695	**0.834**			
2 Ideal L2 self	0.883	0.557	0.317	**0.746**		
3 Enjoyment	0.866	0.617	0.286	0.304	**0.786**	
4 Engagement	0.794	0.562	0.427	0.483	0.455	**0.750**

Bold values indicate the square root of AVE for each construct.

Model fit was evaluated using several commonly reported criteria (Kline, 2023), including the chi-square to degrees of freedom ratio (*χ^2^/df*; < 5), the Root Mean Square Error of Approximation (RMSEA; < 0.10), the Normed Fit Index (NFI; > 0.90), Relative Fit Index (RFI; > 0.90), Incremental Fit Index (IFI; > 0.90), Tucker–Lewis Index (TLI; > 0.90), and the Comparative Fit Index (CFI; > 0.90). The overall measurement model fit the data well (*χ^2^/df* = 1.177, RMSEA = 0.018, NFI = 0.962, RFI = 0.956, IFI = 0.994, TLI = 0.993, CFI = 0.994).

#### 4.1.2 Results of descriptive statistics and correlation analysis

The results of Harman’s single-factor test showed that six factors had eigenvalues greater than 1, and the first factor accounted for 32.422% of the total variance. Since this value was below the commonly recommended threshold of 40%, common method bias was not considered a serious concern in this study (Harman, 1976). Table 2 reports the results of the normality check, along with the descriptive statistics and intercorrelations among the four focal constructs. Univariate normality was supported, as all skewness and kurtosis values fell well below the recommended cutoffs of 2 and 7, respectively (Kline, 2023). Multicollinearity was not a serious concern, as all VIF values were below the recommended threshold of 5 (Hair et al., 2021). The means (all above 3.00) together with moderate standard deviations suggest that participants generally reported relatively high levels of growth mindset, ideal L2 self, enjoyment, and engagement. In addition, all constructs were significantly and positively correlated, providing preliminary support for the hypothesized associations.

**Table 2:** Results of descriptive statistics and Pearson correlation analysis.

Variable	1	2	3	4
1 Growth mindset	1			
2 Ideal L2 self	0.277***	1		
3 Enjoyment	0.252***	0.267***	1	
4 Engagement	0.337***	0.389***	0.370***	1
M	3.766	3.460	3.653	3.755
SD	0.895	0.805	0.753	0.753
Skewness	−0.301	0.097	−0.328	−0.659
Kurtosis	−0.350	−0.201	−0.525	0.541
Tolerance	0.889	0.882	0.894	-
VIF	1.125	1.134	1.118	-

***p < 0.001.

#### 4.1.3 Results of SEM

**Figure 2:**
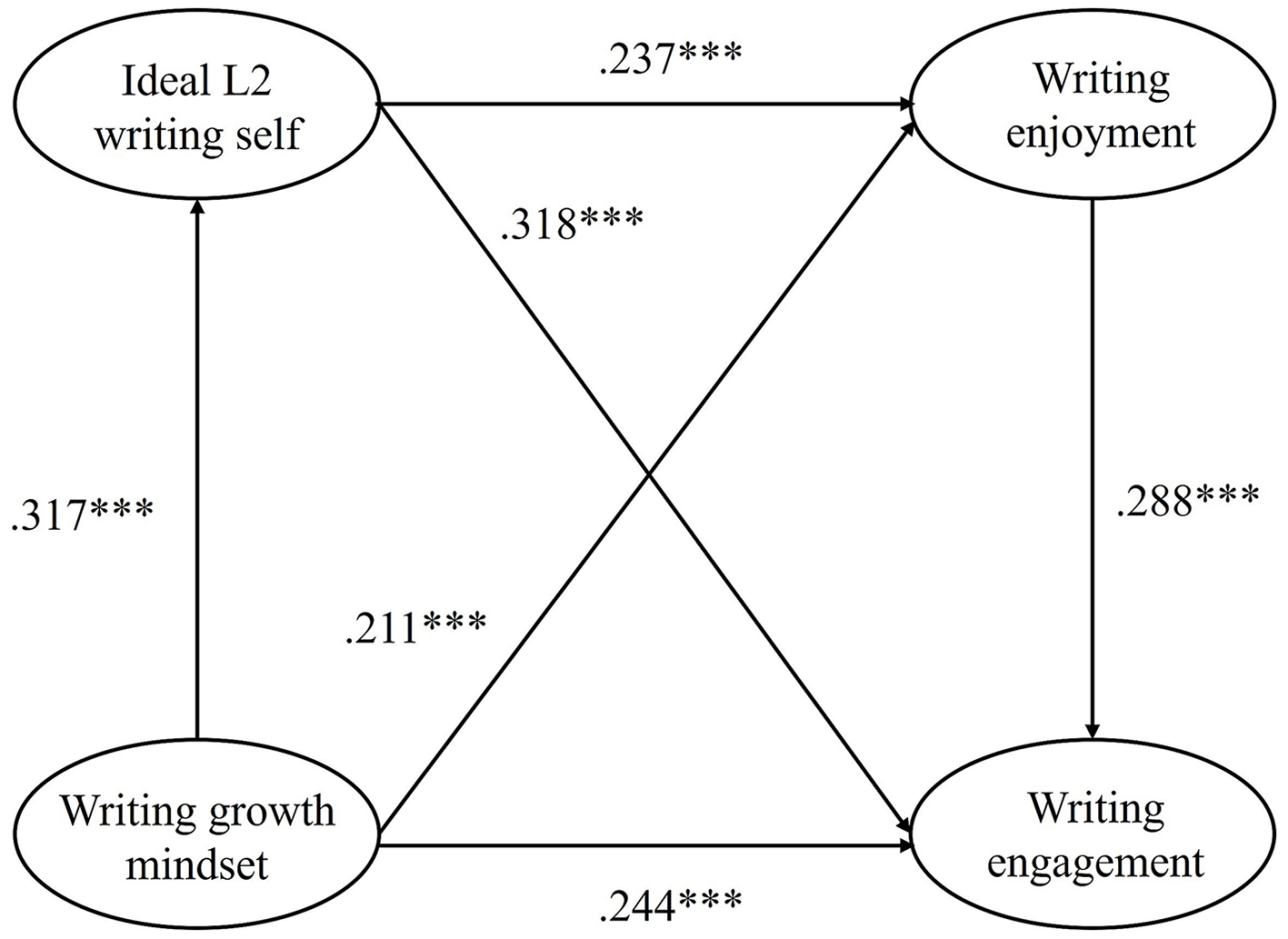
The final structural model. ***p < 0.001.

The hypothesized structural model fit the data well (*χ^2^/df* = 1.177, RMSEA = 0.018, NFI = 0.962, RFI = 0.956, IFI = 0.994, TLI = 0.993, CFI = 0.994), supporting subsequent hypothesis testing. As shown in Table 3 and Figure 2, all specified direct paths were positive and statistically significant, providing support for the proposed direct-effect hypotheses.

**Table 3:** SEM analysis.

Hypotheses	*β*	SE	*p*	Results
H1 Growth mindset → Ideal L2 self	0.317	0.048	***	Supported
H2 Growth mindset → Enjoyment	0.211	0.047	***	Supported
H3 Growth mindset → Engagement	0.244	0.042	***	Supported
H4 Ideal L2 self → Enjoyment	0.237	0.050	***	Supported
H5 Ideal L2 self → Engagement	0.318	0.046	***	Supported
H6 Enjoyment → Engagement	0.288	0.047	***	Supported

***p < 0.001.

To clarify the mechanisms underlying the proposed model, the mediating roles of ideal L2 self and enjoyment, including their sequential mediation, were examined. Indirect effects were evaluated using bias-corrected bootstrapping with 5,000 resamples and 95% confidence intervals (CIs). As reported in Table 4, the indirect effects were significant, including the sequential pathway from growth mindset to engagement via ideal L2 self and enjoyment, as all corresponding CIs excluded zero.

**Table 4:** Results of mediation analysis.

Path	*β*	SE	95% CI		*p*
			Lower	Upper	
Growth mindset → Ideal L2 self → Engagement	0.101	0.022	0.064	0.151	***
Growth mindset → Enjoyment → Engagement	0.061	0.019	0.029	0.107	***
Growth mindset → Ideal L2 self → Enjoyment → Engagement	0.022	0.008	0.009	0.041	***

***p < 0.001.

### 4.2 Qualitative results

#### 4.2.1 Growth mindset as a motivational anchor for sustained engagement

Findings showed that reframing challenges and feedback as learning opportunities plays a central role in sustaining learners’ engagement in AI-mediated EFL writing. Rather than interpreting errors as signs of limited ability, learners tend to view AI-generated feedback as guidance for improvement. As illustrated by P1, multiple AI suggestions are not perceived as evidence of failure but as “small directions” that signal specific areas for development, which encourages further revision instead of withdrawal. Similarly, P4 explicitly contrasts past frustration with a current understanding that revision is part of an ongoing process, suggesting a shift toward a growth-oriented interpretation of writing development. These accounts indicate that learners’ reinterpretation of feedback reduces emotional threat and supports continued effort across iterative cycles. Together, the interview data demonstrate that when challenges are construed as developmental opportunities, learners are more likely to remain actively engaged even in demanding revision processes. Typical interview excerpts are presented below.

P1: *When I see many suggestions from the AI, I do not feel that I’m bad at writing*. *I think it just shows what I still need to improve*. *Each comment feels like a small direction telling me where to go next, so I prefer to revise again instead of stopping.*

P4: *Before, too many corrections would make me frustrated. Now I feel they are normal. Writing is a process, and every revision means I’m closer to a better version. That idea makes me continue even when it’s tiring*.

#### 4.2.2 Ideal L2 self as a motivational anchor for sustained engagement

Findings showed that learners’ ideal L2 self functions as a motivational anchor that supports sustained engagement in AI-mediated EFL writing. Learners frequently connect their current writing practices with an envisioned future identity as more competent English users. For example, P2 explicitly links revision effort to a desired future self who can write more confidently, indicating that this imagined identity provides direction and persistence in writing tasks. Likewise, P12 emphasizes that writing extends beyond completing assignments, reflecting a self-driven aspiration to “become someone who can use English well,” which motivates repeated checking and refinement of sentences. These accounts suggest that ideal L2 self transforms writing from a task-based requirement into a goal-directed activity embedded in personal development. In this way, engagement is sustained not merely by external demands but by alignment with learners’ internalized future self-guides. Typical interview excerpts are presented below.

P2: *I hope that in the future I can write English more confidently. When I revise my essays, I always think about that version of myself. It reminds me why I should keep working on my writing, even if it takes a lot of time*.

P12: *I do not only write for this assignment. I write because I want to become someone who can use English well. That idea makes me willing to check my sentences again and again*.

#### 4.2.3 Enjoyment as an affective driver of continued engagement

Findings showed that enjoyment operates as an affective driver that supports continued engagement in AI-mediated EFL writing. Learners describe positive emotions emerging from visible improvement and interactive feedback processes. As P14 notes, noticing that a revised version is better than the previous one generates a sense of achievement, which reinforces willingness to continue revising. Similarly, P20 highlights the interactive quality of AI-supported writing, describing it as a communicative experience that reduces boredom and sustains effort. These statements suggest that enjoyment is closely tied to both perceived progress and task interactivity, which together enhance learners’ emotional investment in writing. Rather than being a peripheral experience, enjoyment appears to function as an affective resource that supports ongoing participation across revision cycles. Typical interview excerpts are presented below.

P14: *When I see my new version is better than the old one, I feel happy. It gives me a sense of achievement, and I want to continue revising to make it even better*.

P20: *Using AI makes writing more interesting. It feels like someone is communicating with me. Because of that, I do not feel bored, and I’m willing to keep working on my essay*.

## 5 Discussion

### 5.1 Interpreting the quantitative findings in AI-mediated EFL writing

Given that this study was situated in AI-mediated EFL writing among ethnic minority EFL learners in China, the following discussion interprets the findings in relation to this specific learning context and learner population. Rather than treating the results as general evidence for all EFL learners, the discussion focuses on how growth mindset, ideal L2 self, and enjoyment may support engagement under the educational, linguistic, sociocultural, and technological conditions that shape ethnic minority learners’ writing experiences. This orientation is consistent with the direction established in the Introduction, where AI-mediated writing and ethnic minority learner experiences were identified as the key contextual focus of the study. Accordingly, the discussion highlights not only the statistical relationships among the variables, but also their meaning for understanding sustained engagement in AI-supported writing and iterative revision among the participants in this study.

First, the present study found that growth mindset was positively related to ideal L2 self, enjoyment, and engagement in AI-mediated EFL writing. This suggests that learners who believe their writing ability can be developed are more likely to experience greater alignment with their ideal L2 self, higher enjoyment, and increased engagement. This finding is consistent with SCT, which posits that personal factors influence behavior and are interrelated (Bandura, 1986, 1997). These results align with previous research showing that learners with a stronger growth mindset are more likely to envision themselves as competent L2 users (Fathi et al., 2024; Yao et al., 2024), and interpret challenges and feedback as manageable and supportive rather than threatening, fostering positive emotional experiences (Xu, 2025; Yang and Wu, 2025). Additionally, growth-oriented learners exhibit greater persistence, adaptive strategies, and task involvement in language learning contexts (Jiang et al., 2024; Vestad and Bru, 2024). This study extends the existing literature by confirming that this relationship holds in the context of AI-mediated EFL writing, where learners frequently encounter automated feedback and repeated revision tasks, thus emphasizing the role of mindset in sustaining ideal L2 self, enjoyment, and engagement over time. This suggests that, for the ethnic minority learners in this study, a growth mindset may be particularly important in helping them interpret AI-generated feedback as developmental support rather than as evidence of limited writing ability.

Second, the present study found that ideal L2 self was positively related to enjoyment and engagement in AI-mediated EFL writing. This indicates that learners with a stronger ideal L2 self are more likely to experience higher enjoyment and increased engagement during their writing tasks. This finding aligns with SCT, which posits that personal factors, such as self-guides, influence behavior and emotional responses (Bandura, 1986, 1997). This finding is consistent with prior research suggesting that self-guides and personal goals are closely associated with positive emotions in foreign language learning (Fathi and Hejazi, 2024; Zhao et al., 2023). Moreover, this finding supports findings from the L2 motivational self system, which has consistently shown that a stronger ideal L2 self is linked to greater intended effort, persistence, and active participation in language learning (Sun et al., 2024; Zhu et al., 2023). The present study extends this line of research by demonstrating that ideal L2 self is similarly associated with enjoyment and engagement in AI-mediated EFL writing contexts, highlighting the significance of personal goals in fostering motivation and emotional involvement in AI-supported learning environments. For the ethnic minority learners in this study, ideal L2 self may therefore help connect AI-mediated writing tasks with future academic, professional, and intercultural aspirations.

Third, enjoyment is positively associated with engagement in AI-mediated EFL writing. This suggests that learners who find enjoyment in their writing tasks are more likely to stay engaged and invest effort in the learning process. This finding aligns with SCT, which posits that emotional experiences, such as enjoyment, can motivate behavior and influence sustained engagement in tasks (Bandura, 1986, 1997). This interpretation is consistent with prior research showing that enjoyment is closely associated with greater behavioral participation, deeper cognitive involvement, and stronger willingness to persist in language learning tasks (Liu, 2022; Ünsal-Görkemoğlu and Karacan, 2024). The present findings extend this line of research by demonstrating that these associations remain salient in AI-mediated EFL writing contexts, where learners engage with automated feedback and revision processes. This highlights the importance of enjoyment in sustaining engagement in AI-supported writing environments. In this context, enjoyment may emerge from visible improvement, interactive feedback, and the sense that revision becomes more manageable with AI support.

Fourth, in addition to these direct relationships, the present study identified several mediation relationships. Specifically, growth mindset was found to influence engagement through ideal L2 self, enjoyment, and through both ideal L2 self and enjoyment. This suggests that learners with a growth mindset are more likely to develop a stronger ideal L2 self and greater enjoyment, which, in turn, fosters increased engagement in AI-mediated EFL writing tasks. This is among the first studies, to our knowledge, to examine these mediation relationships in the context of AI-mediated EFL writing. These findings align with SCT, which posits that personal factors, such as mindset and self-concept, influence emotional experiences and behavior (Bandura, 1986, 1997). Moreover, the results extend the L2 motivational self system, which has shown that learners’ ideal L2 self can mediate the relationship between personal beliefs and engagement (Dörnyei, 2009). By revealing how growth mindset influences engagement indirectly through self-related and emotional factors, this study highlights the complex, interrelated processes that shape engagement in AI-supported EFL writing environments. These mediation findings respond to the Introduction by showing how belief-related, self-related, and affective resources jointly support engagement in AI-mediated EFL writing among ethnic minority EFL learners.

### 5.2 Learners’ experiences of engagement in AI-mediated EFL writing

The qualitative findings further showed how ethnic minority EFL learners experienced engagement in AI-mediated EFL writing through their responses to feedback, future self-guides, and enjoyment during revision. Learners’ engagement in AI-mediated EFL writing was closely shaped by how they responded to challenges and feedback during the writing process. Many learners described that they gradually learned to interpret AI-generated feedback as guidance for improvement rather than as evidence of poor ability. This shift in interpretation helped reduce feelings of threat and made revision feel more manageable. Instead of withdrawing when encountering multiple suggestions, learners tended to view each comment as a direction for further development, which encouraged continued effort. Such responses indicate that engagement is sustained not simply by receiving feedback, but by how learners make sense of it. When feedback is understood as part of a normal developmental process, learners are more willing to persist across iterative revision cycles (Jiang et al., 2024; Vestad and Bru, 2024). In this way, engagement emerges from an active process of interpreting, coping with, and responding to difficulties. These findings suggest that learners’ engagement in AI-mediated writing is closely tied to their evolving ways of dealing with challenges and feedback.

Learners’ engagement was also shaped by how they connected current writing activities to their imagined future selves as English users. Many learners explained that they did not view writing merely as completing assignments, but as a step toward becoming more confident and competent in English. This future-oriented perspective gave personal meaning to writing tasks and made sustained effort feel worthwhile. When learners envisioned themselves as proficient L2 users, they were more inclined to invest time in polishing language, refining ideas, and revising drafts. Engagement, therefore, was experienced as a goal-directed process rather than a task-bound obligation (Sun et al., 2024; Zhu et al., 2023). Such accounts indicate that learners remain engaged when writing is perceived as contributing to long-term personal development. In AI-mediated EFL writing, where progress can be repeatedly observed through revision, this connection to future selves appears to strengthen learners’ willingness to stay involved.

Learners further described enjoyment as an important part of their engagement in AI-mediated EFL writing. Many reported feeling satisfied when they noticed that revised versions of their texts were clearer or more natural than previous drafts. These positive emotional experiences made writing feel more interesting and less burdensome, which encouraged continued participation. Learners also noted that interacting with AI tools created a sense of dialogue, making the writing process more dynamic. Enjoyment, therefore, emerged not as a constant state, but as something that developed through noticing improvement and receiving useful feedback (Liu, 2022; Ünsal-Görkemoğlu and Karacan, 2024). When learners experienced such positive feelings, they were more willing to continue revising and refining their work. These accounts suggest that engagement is sustained, in part, through the accumulation of enjoyable moments during iterative writing and revision.

### 5.3 Theoretical and pedagogical implications

This study offers several theoretical implications for understanding engagement in AI-mediated EFL writing. First, by integrating growth mindset, ideal L2 self, and enjoyment within a single explanatory framework, the study advances a more comprehensive account of engagement as the outcome of interacting personal factors rather than as a purely behavioral manifestation. Second, the findings extend SCT into AI-mediated EFL writing contexts by demonstrating how learners’ belief systems, future-oriented self-guides, and affective experiences jointly shape their writing behaviors in technology-rich environments. In line with the principle of triadic reciprocal determinism, engagement is conceptualized as emerging from the interaction among personal beliefs, writing behaviors, and AI-mediated environmental feedback. Third, the study highlights the central role of self-regulatory processes, showing how growth mindset and ideal L2 self can influence learners’ persistence and emotional experiences during iterative revision. Collectively, these contributions refine engagement research by situating it within the SCT framework that emphasizes person–environment interactions in AI-mediated EFL writing contexts.

The findings also yield important pedagogical implications for AI-mediated EFL writing instruction. Teachers should support learners in interpreting AI-generated feedback as a developmental resource rather than as a judgment of ability, thereby fostering more adaptive responses to revision. In addition, instruction can encourage learners to connect writing tasks with their long-term language goals and future identities, helping them perceive writing as meaningful beyond immediate assessment requirements. Teachers may also design activities that make progress visible and emphasize positive experiences during drafting and revision, such as highlighting improvements across versions or encouraging reflection on learning gains. Importantly, these implications suggest that AI tools alone do not automatically enhance engagement; rather, engagement depends on how instructional practices activate learners’ beliefs, self-guides, and emotions. Purposeful pedagogical design is therefore essential for maximizing the potential of AI-mediated writing.

## 6 Conclusion

This study investigated the psychological mechanisms underlying engagement in AI-mediated EFL writing among ethnic minority EFL learners in China by examining growth mindset, ideal L2 self, and enjoyment. Using a mixed-methods design, the quantitative findings revealed significant associations among learners’ beliefs, motivational self-guides, emotional experiences, and engagement, while the qualitative findings further illustrated how learners interpreted AI-generated feedback, connected writing to future selves, and experienced enjoyment during iterative revision. Together, these results suggest that engagement in AI-mediated EFL writing is sustained through an interplay of cognitive, motivational, and affective processes rather than through technological affordances alone. In response to the research gap identified in the Introduction, the specific contribution of this study lies in showing how growth mindset, ideal L2 self, and enjoyment function as linked psychological resources that support sustained writing engagement in a technology-rich learning environment. By situating these mechanisms in the experiences of ethnic minority EFL learners, this study provides a more context-sensitive understanding of engagement in AI-mediated EFL writing and underscores the importance of attending to learners’ psychological resources when designing AI-mediated writing instruction.

Several limitations of this study should be acknowledged. First, although ethnic minority EFL learners were the focus of this study, the sample was limited to particular regional and institutional contexts, which may constrain the transferability of the findings. Future research could include ethnic minority learners from broader regions and varied institutional settings. Second, the cross-sectional design does not capture changes in learners’ engagement or psychological factors over time. Longitudinal or experience-sampling designs would provide deeper insight into developmental trajectories. Third, AI-mediated EFL writing was broadly operationalised in this study. Specific tool types, use frequency, teacher guidance, and depth of AI involvement were not systematically recorded. Future studies should examine these differences to better capture variation in students’ AI-supported writing experiences. Moreover, ethnic minority EFL learners should not be treated as a homogeneous group. Future research could compare subgroups with different ethnic, linguistic, regional, and educational backgrounds to better understand variation in their AI-mediated EFL writing experiences.

## Data Availability

The raw data supporting the conclusions of this article will be made available by the authors, without undue reservation.
